# Performative updates and the modeling of speech acts

**DOI:** 10.1007/s11229-023-04359-0

**Published:** 2024-01-12

**Authors:** Manfred Krifka

**Affiliations:** Leibniz-Zentrum Allgemeine Sprachwissenschaft (ZAS) and Humboldt-Universität zu Berlin, Pariser Str. 1, 10719 Berlin, Germany

**Keywords:** Speech acts, Assertions, Declarations, Dynamic interpretation, Explicit performatives

## Abstract

This paper develops a way to model performative speech acts within a framework of dynamic semantics. It introduces a distinction between performative and informative updates, where informative updates filter out indices of context sets (cf. Stalnaker, Cole (ed), Pragmatics, Academic Press, 1978), whereas performative updates change their indices (cf. Szabolcsi, Kiefer (ed), Hungarian linguistics, John Benjamins, 1982). The notion of index change is investigated in detail, identifying implementations by a function or by a relation. Declarations like *the meeting is (hereby) adjourned* are purely performative updates that just enforce an index change on a context set. Assertions like *the meeting is (already) adjourned* are analyzed as combinations of a performative update that introduces a guarantee of the speaker for the truth of the proposition, and an informative update that restricts the context set so that this proposition is true. The first update is the illocutionary act characteristic for assertions; the second is the primary perlocutionary act, and is up for negotiations with the addressee. Several other speech acts will be discussed, in particular commissives, directives, exclamatives, optatives, and definitions, which are all performative, and differ from related assertions. The paper concludes a discussion of locutionary acts, which are modelled as index changers as well, and proposes a novel analysis for the performative marker *hereby.*

## Introduction

On the evening of November 9, 1989, at the press conference of the Central Committee (ZK) of the Socialist Union Party (SED) of the German Democratic Republic (DDR), the newly nominated Secretary of Information, Günther Schabowski, was asked by an Italian journalist whether he considered it a mistake that the government was planning to liberalize emigration for its citizens. Schabowski replied that, according to his knowledge, the new rulings were published already. He got out a document that he had received just an hour before at the meeting of the ZK, at which he had arrived late, and read its text, which started that citizens could apply for permissions to travel abroad without any conditions, such as visits of relatives. When asked when this new ruling would become effective, Schabowski said, immediately, according to his knowledge (actually, the ZK planned to make the new ruling public the following day, November 10, and did not want the document distributed before that). One hour later, thousands of East Berliners had stormed the Berlin wall and found themselves in West Berlin for the first time in their life.

Schabowski’s role, as Secretary of Information, was to *inform* the public about the decisions of the ZK. The role of the ZK was to *make* these decisions. Schabowski overstepped his authority by stating that the borders would be open immediately, but this part of his message was also meant as information, not as a decision by his own – hence his hedge, “according to my knowledge”.

This is the classical distinction of Austin (1962) between *constative* and *performative* speech acts. It also figures prominently in the classification of Searle (1976), who distinguishes these speech acts as *representatives* and *declarations*. According to Searle, with representatives, speakers adjust the words to the world, whereas with declarations, they also adjust the world to the words. Declarations, for Searle, are the central tool to create the fabric of the social world, and hence of crucial importance for the human condition (cf. Searle, 2010).

While the distinction between constatives and performatives is a central tenet of classical speech act theory, it does not figure prominently in model-theoretic semantics. Dynamic models of communication in the tradition of Hamblin (1971), Stalnaker (1978), Kamp (1981), Heim (1983), Rooth (1987) and Groenendijk and Stokhof (1990) focused on modeling the update of the common ground with *information about* the world. One exception within model-theoretic semantics is Szabolcsi (1982), who proposed an extension of Montague (1973) for explicit performatives like promises. According to Szabolcsi, such speech acts are not interpreted as true or false with respect to a given index, they rather change that index. In a similar fashion, Lascarides & Asher (2003), working within the general setting of discourse representation theory, proposed that imperatives change the interpretation of the world at which DRSs are interpreted.

In Sect. 2, I will motivate the need to distinguish between informative and performative updates in detail. In Sect. 3 I will have a closer look at Szabolcsi’s notion of the change of an index to satisfy a proposition. Section 4 gives an interpretation of declarations within this setting, i.e. of implicit performatives like *the meeting is (hereby) adjourned* and of explicit performatives like *I (hereby) declare that the meeting is adjourned*. Section 5 discusses the tense forms of declarations. In Sect. 6 I contrast declarations with assertions like *the meeting is (already) adjourned*, which are analyzed as having a performative part in which the speaker gives a guarantee for the truth of the proposition, and an informative part in which this proposition itself is added to the common ground. Section 7 is a short overview of other speech acts for which modeling in terms of performative updates is plausible, in particular commissives, directives, expressives, and definitions. In Sect. 8 I propose a model of the locutionary act, the utterance itself, in terms of (a series of) performative updates. And in Sect. 9 I propose a novel theory of the adverbial *hereby* that marks declarations, and which can also explain the occurrence of *hereby* in embedded clauses.

## Modeling informative vs. performative update

### Stalnaker and Szabolcsi

The notion of *Common Ground*, as the body of information that the participants of a conversation assume to be shared at a given time, has turned out to be extremely useful for our understanding of communication, both in formal modeling and in our analysis of particular conversations (cf. Clark, 1996; Stalnaker, 1978, 2002). Also, this concept of Common Ground can be related to the notions of shared attention and intention, arguably the most distinctive trait of human cognition even beyond language (Tomasello et al., 2005).

An adequate notion of Common Ground contains several dimensions that relate to the shared attention on the situation in which the exchange takes place, the shared background knowledge of the participants, the information that has been exchanged and the discourse referents that have been introduced so far during the conversation, as well as assumptions about the individual bodies of knowledge and attitudes of the participants (cf. e.g. Ginzburg, 2012). The model I will work with here is kept deliberately simple, to concentrate on the difference between constative and performative aspects of utterances. In particular, the Common Ground is modelled by *context sets*, i.e. sets c of world-time indices i that represent the ways how the world could be like at the current time, according to the information that the participants assume to be shared (Stalnaker, 1978).

According to this extremely simple model of Common Ground, informing that a proposition φ is true simply restricts an input context c to an output context set c′:

1. Informative update:
  $$\begin{aligned} {\text{c}} + {\text{inform}}\left( \varphi \right) &= \{ {\text{i}} \in {\text{c }}| \, \varphi \left( {\text{i}} \right)\} = \left\{ {{\text{i }}|{\text{ i}} \in {\text{c }} \wedge \, \varphi \left( {\text{i}} \right) = {\text{true}}} \right\} = {\text{c}}^{\prime}\hfill \\ \end{aligned}$$

This captures what Austin (1962) called *constative* or *descriptive* use of language, i.e. representatives, in particular, assertions. It does not apply to *performative* utterances like directives, commissives and declarations. Such speech acts do not inform the addressee about what the actual world-time index is like, but rather change this index. With directives, the speaker creates an obligation for the addressee; with commissives, the speaker creates a self-obligation; and with declarations, the speaker creates a new fact by his or her utterance.

Dynamic semantics has been extended to certain types of non-informative discourse. For example, Portner (2004) proposed that the state of a conversation should not only contain a common ground, as a representation of factual information, but also a set of question meanings that capture the open issues of a conversation, and a to-do-list, a function from individuals to sets of propositions that captures the obligations of participants. Just as assertions add to the factual knowledge (which Portner represents by a set of propositions), questions enrich the question set, and commands enrich the to-do-lists.^1^

A quite different approach to performative speech acts was proposed by Szabolcsi (1982). Developed independently from dynamic semantics and the work of Stalnaker, it proposes a type of update to model performative speech acts within Montague Grammar. The basic idea is that a performative speech act is interpreted as a “transition from one state of affairs to another” (Szabolcsi, 1982, cf. also Sbisà, 2002 for the idea of speech acts as context changers). In Montague Grammar, sentences are interpreted as true or false with respect to a given model. Szabolcsi suggests that performative utterances enact a *change* of the model itself, as the representation of the world. For this, she proposes a function that changes a world-time index i to an index i′ with i ≤ i′ (that is, i′ is equal or later than i), where the index i′ is identical to i with the (possible) difference that φ(i′), i.e., that φ is true at i. While Szabolcsi writes i[φ] for this index i′, we will use the notation i + φ for better comparison with the informative update. We call this *functional index change*^2^:

(2) Functional index change (Szabolcsi):
  $$\begin{aligned} & {\text{i}} + \varphi = \iota {\text{i}}^{\prime}[{\text{i}} \le {\text{i}}^{\prime}\, \wedge {\text{ i}}^{\prime}{\text{ is identical to i with the possible exception that }}\varphi \left( {{\text{i}}^{\prime}} \right)]. \hfill \\ \end{aligned}$$

In case φ is true at i already, we have i + φ = i, i.e. functional update does not change anything.^3^ Functional update may be restricted in case the index i cannot develop into one in which φ is true, making it a partial function. Index change may be restricted in other ways, e.g. capturing the felicity conditions for speech acts that have to be satisfied (cf. Searle, 1976). Also, the indices change due to the locutionary act of uttering an expression; this aspect will be addressed in Sect. 8.

Szabolcsi’s update can be integrated into a model of context set update as changes of the individual indices of the context set:

(3) Performative update:
  $$\begin{aligned} {\text{c}} + {\text{perform}}\left( \varphi \right)& = \{ {\text{i}} + \varphi \, |{\text{ i}} \in {\text{c}}\} \hfill \\& = \{ {\text{i}}^{\prime}\, | \, \exists {\text{i}}\left[ {{\text{i}} \in {\text{c }} \wedge {\text{ i}} \le {\text{i}}^{\prime}\, \wedge {\text{ i}}^{\prime}{\text{ is identical to i except for }}\varphi \left( {{\text{i}}^{\prime}} \right)} \right]\} \hfill \\ & = {\text{c}}^{\prime}\hfill \\ \end{aligned}$$

Performative update does not remove indices of the context set c but changes them minimally so that the proposition φ holds. While c + inform(φ) is always a subset of c, c + perform(φ) is not generally a subset of c, except for those cases in which φ is established throughout c already.^4^

It might be questioned (as one reviewer did) whether we need different update mechanisms to model constative vs. performative speech acts. After all, we end up with a context set at which the proposition φ is true, irrespective of the ways how this has been achieved. I would like to argue that we need distinct updates when we want to capture the essential differences between the two types of speech acts. Consider the situation on November 9, 1989, again. It was common knowledge that citizens of the GDR could not apply for travel abroad. If a non-authorized person utters (4) in this situation, it would be considered a lie. If the ZK utters (4), however, in its capacity to change the law, this is not a lie but makes the proposition true.

(4) *As of now, citizens can apply for travel abroad.*

Indeed, one cannot lie with a performative speech act, or even assert a falsehood unwillingly (cf. Marsili 2021). As already observed by Austin (1961), performatives cannot be true or false to begin with. Also, a reaction requesting evidence like *Who says so?* is not appropriate for performative utterances, whereas a reaction like *Don’t do that!* is fine for performatives but odd for assertions.

The need to distinguish between informative and performative updates may be questioned for declarations because they are expressed by the same sentence grammatical type, declarative sentences. However, there are subtle differences: Performative sentences allow for the optional use of *hereby*, and they cannot be hedged with epistemic or evidential operators such as *certainly*, *could* or *apparently*, as observed by Austin (1961). In Japanese, there are a number of optional sentence-final particles that generate performative (or informative) interpretations, cf. Kubo (1992). In German, the verbal mood Konjunktiv I can be used to express performative utterances, as in *Es* *werde* *Licht* ‘Let there be light’, cf. Krifka (2023).

One could argue that a declaration like (4) is not a performative update of the current indices, but an informative update about the immediately following world-time indices. We could model this in the following way: For every index i that is part of the input context set c, φ is false, but there are indices i′ whose time component is a moment later than i for which φ is true, and the set of these indices i′ is the output common ground. The performative utterance in (4) would then amount to the assertion of a future proposition as in (5).

(5) *Citizens will apply for travel abroad.*

However, such constative utterances can be false, whereas performative utterances can only be infelicitous, not false. Also, we would have to explain why performative utterances of sentences such as (4), in spite of their present tense, are interpreted as future-oriented.

Explicit performatives are sometimes analyzed as a subspecies of assertions, self-satisfying assertions (cf. e.g. Bach & Harnish, 1979). The idea is as follows: An utterance of *I order you to sign the report*, understood as an assertion, commits the speaker to the truth of the proposition ‘speaker orders addressee to sign the report’. From this it follows that the speaker has both the power and the intention to have the report signed. This argument was criticized by Searle (1989), who argues that from the commitment to the truth of this proposition, the power and intention does not follow. It was defended against this criticism by Condoravdi and Lauer (2011), who argue that it follows from the truth of the proposition ‘speaker orders addressee to sign the report’ that the speaker has the power and intention. However, this discussion – as well as Eckardt (2012) – focuses on explicit performatives that name the speech act that is performed, such as *order*, *claim* or *ask*. The arguments do not obviously extend to implicit performatives like *the meeting is (hereby) adjourned.*

I take it that it is a desideratum to come up with a theoretical account that distinguishes between assertions and declarations, or between informative and performative updates.

## The notion of index change

The account of performative updates proposed by Szabolcsi (1982) hinges on the question whether index change, as defined in (2), is a viable notion. Recall that Szabolcsi requires that there is, for an index i, a *unique* index i′ with i ≤ i′ such that i′ differs from i *minimally* insofar as φ is true at i′. In this section we will have a closer look at the conditions under which these conditions can be satisfied.

One problem of Szabolcsi’s definition of index change (2) is that it excludes independent cotemporaneous changes. Take as an example that S utters to A at the index i, *I congratulate you.* Szabolcsi models this as the functional update with i + ‘S has congratulated A’, which is the index i′ with i ≤ i′ that is similar to i except that at i′, the effect of the congratulation of a by s has taken effect. The similarity condition will force i′ to be as temporally close to i as possible – otherwise, other events would have happened in between, and i and i′ would be more different. In a discrete temporal structure, i′ will be an index immediately following i. But we do not want to exclude that other changes happen at precisely the same time that have nothing to do with the congratulation, changes that went unnoticed by the participants or that are part of their joint attention. For example, we would not like to exclude that at the time S says *I congratulate you*, A sneezes.

One way to avoid this problem of independent changes is within a framework of branching time,^5^ following Prior (1967), Rescher and Urquardt (1971) and Thomason (1984). We assume a transitive relation < on the set of indices I with the condition of backwards linearity, i.e. it holds for all i, i′, i″ ∈ I that if i′ < i and i″ < i, then either i′ = i″ or i′ < i″ or i″ < i′. This entails that for each index i, the past is fixed, and the future is open. We now define functional index change as follows:

(6) Functional index change with respect to a temporal order <:
  i + φ is the unique index i′ such that
  - for all i″, i″<i ↔ i″<i′
  - φ(i′) = 1
  - i and i′ do not differ in any other relevant proposition except φ.

(6)(a) guarantees that i and i′ have the same predecessors, (b) states that the proposition φ is true for the changed index i′, and (c) ensures that i and i′ differ in no other respect. Notice that in case φ(i) = 1, i and i′ are identical following (c), that is, no index change has occurred. In the crucial case where φ(i) = 0 and φ(i′) = 1, the indices i and i′ are not ordered by < ; rather, an instance of branching has occurred with φ(i) = 0 and φ(i′) = 1. Different from Szabolcsi (1982), in this case the changed index i′ with i′ = i + φ is not after i within the same history; rather, i and i′ are the beginning points of new histories. In a sense, i and i′ can be defined as cotemporaneous, where we define the time of indices across histories following of Di Maio & Zanardo (1994) as a mapping τ from indices I that are partially ordered by < onto times T that are linearly ordered by ≺. This function has the following properties:

(7) Mapping indices to times by τ:
  $$\begin{aligned} & {\text{a}}. \,\,{\text{For all i}},{\text{ i}}^{\prime}\, \in \,{\text{I}}:{\text{ If for all }}i^{\prime \prime} ,{\text{ }}i^{\prime \prime} \, < \,{\text{i}}\, \leftrightarrow \,{\text{i}}^{\prime \prime} \,< \,{\text{i}}^{\prime} ,{\text{ then }}\tau \left( {\text{i}} \right)\, = \,\tau \left( {{\text{i}}^{\prime} } \right). \hfill \\& {\text{b}}.\,\, {\text{If i}}\, < \,{\text{i}}^{\prime} {\text{ then }}\tau \left( {\text{i}} \right) \, \prec \, \tau ({\text{i}}^{\prime} ). \hfill \\ \end{aligned}$$

Here, (7)(a) states that i and i′, even when starting different histories, represent the same time. And (b) imposes that the order of times is aligned with the temporal order of indices.

The definitions apply to both dense and discrete orders, but it can be more easily visualized for the discrete case. Example (8) shows seven indices, represented by dots, and the successive times t_n-1_, t_n_ and t_n + 1_ that they are mapped to by τ. It highlights four propositions φ, ¬φ, ψ and ¬ψ that obtain at these indices. Notice that the functional index change i + φ is momentaneous in that it does not take time; we have τ(i) = τ(i′) = t_n_. Notice also that the analysis assumes that the two indices can be distinct even though they satisfy the same propositions, as in the uppermost line, where the indices just differ in their time.

(8) Functional index change: i + φ = i′ in a discrete model
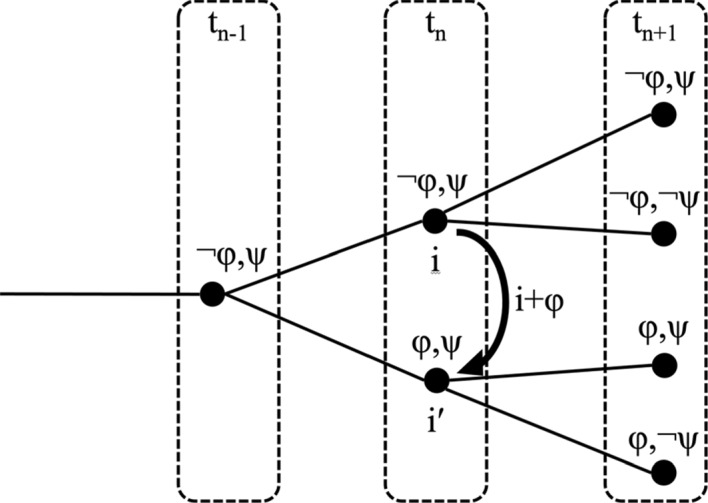

The crucial question now is: How can we work out condition (6)(c)? What does “any other relevant proposition but φ” mean?

First, there are propositions π that follow from φ. These propositions may be entailed by φ logically, such as if π is [φ ∨ φ′], or it might be that it has been established in the current branch that necessarily, whenever φ is true, π is true as well. This situation does not have to concern us. Assume that there is an index i″ with i″ < i such that for all i‴ with i″ ≤ i‴ it holds that φ(i‴) → π(i‴). The functional index change i + φ will result in an index i′ for which not only φ is true but π is true as well, as illustrated in (9).

(9) Functional index change with dependent index change:
  i +φ = i′, where φ → π is established throughout the current histories

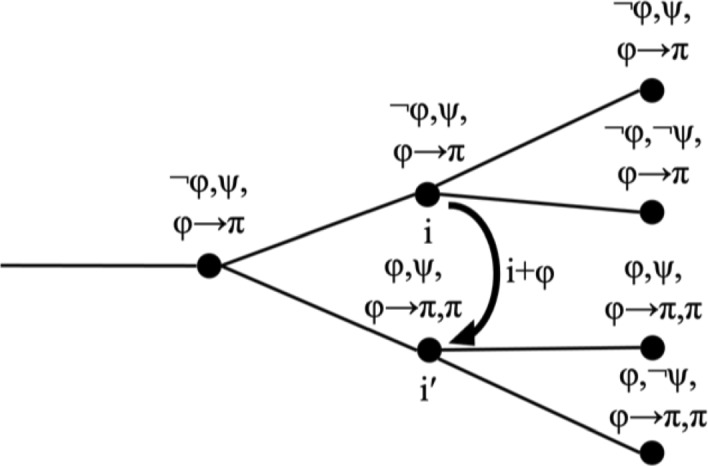


Second, it might happen that with the change from i to i + φ, there is another, independent change. Assume that at the indices before i, both φ and ψ are false, and that ψ happens to become true at index i. The index change i′ = i + φ will keep ψ as true, as illustrated in (10). Compared to the predecessor of i′, both φ and ψ have become true, but only the first change was triggered by the performative update i + φ, the second change is independent of it.

(10) Functional index change with independent index change
  i + φ = i′, with cotemporaneous change from ¬ψ to ψ.
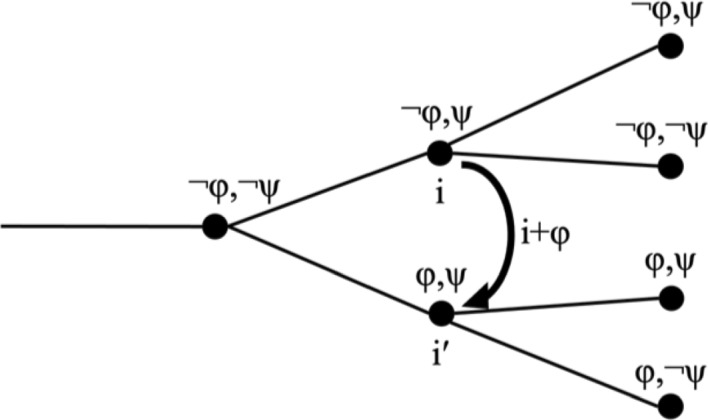

Another case to consider are situations in which there are multiple ways to make a proposition φ true. For concreteness, take φ = λi[π(i) ∨ π′(i)], for which we write [π ∨ π′]. Then the change i′ = i + φ requires that φ(i′) = [π ∨ π′](i′) = 1, but leaves it open whether π or π′ is true at i′. If we want to retain the idea that index changes are *functions*, i.e. right-unique relations, then we have to allow that propositions can be undetermined at particular indices. That is, we have to work with indices are underspecified, similar to situations in the sense of Barwise and Perry (1981) and Kratzer (1989).

(11) Functional index change with a proposition [π ∨ π′],
  where * denotes indeterminacy, with further change to π and π′.
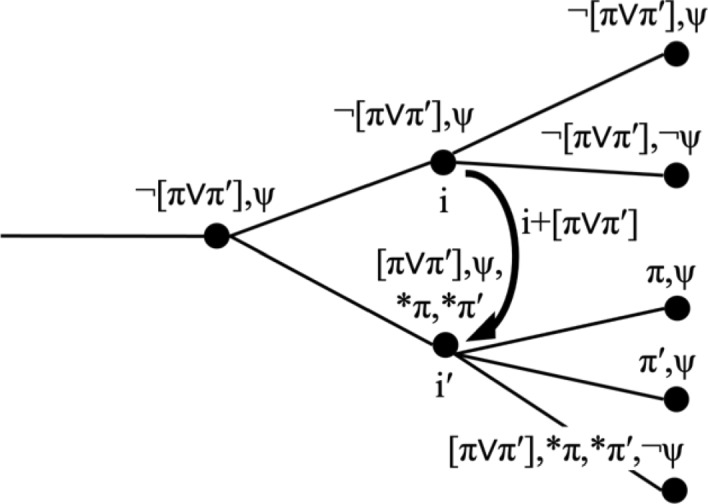

The notation for indeterminacy suggests an approach with a three-valued logic. However, such logics generally exclude that a disjunction is true when both disjuncts are undefined. But this situation is characteristic for disjunction in quantum logic, for which it is essential to model that it is known that a particle is in one state or another, without knowing in which state it is (cf. Aerts et al., 2000).

This configuration also corresponds to the general modelling of information gain, in particular, to updates of Stalnakerian context sets. For example, it is possible that [π ∨ π′] is established throughout c, i.e. ∀i ∈ c[π(i) ∨ π′(i)], but that neither π nor π′ are established in c yet. After all, assuming a *set* c of indices i that fully determine the truth value of all propositions is nothing but a way to express information states that determine them only *partially*. Consequently, once we allow that indices determine the truth value of propositions only partially, we can represent information states by single indices i, instead of sets of indices c. We could still distinguish between the two types of updates by requiring that performative update of i with φ requires that φ is false at i whereas informative update requires that φ is undetermined at i (cf. the discussion below, in (16)).

Another option for dealing with the problem of multiple ways of satisfying a proposition is to retain classical indices that determine the truth value of all propositions, and assume that index change is a relational, not a functional notion. This was proposed in Krifka (2014). To illustrate, we can define i + φ as the *set* of indices i′ that are minimally different from i, as follows:

(12) Relational index change:
  $$\begin{aligned} & {\text{i}}^{\prime} \, \in \,{\text{i}}\, + \,\varphi {\text{ iff}}. \hfill \\& {\text{a}}.\,{\text{for all }}i^{\prime \prime} ,{\text{ }}i^{\prime \prime} \, < \,{\text{i}}\, \leftrightarrow \,{\text{i}}^{\prime \prime} \, < \,{\text{i}}^{\prime} \hfill \\& {\text{b}}.\,\varphi \left( {{\text{i}}^{\prime} } \right)\, = \,{1}. \hfill \\& {\text{c}}. \,{\text{i and}} \, \hbox{i}^\prime {\text{ do not differ in any other relevant proposition but }}\varphi . \hfill \\ \end{aligned}$$

Here, (12)(c) has to be spelled out as in the (A) clause above, and there is no corresponding (B) clause. Example (13) illustrates relational index change.^6^

(13) Relational index change: i+[π ∨ π′], where π, π′ are mutually exclusive
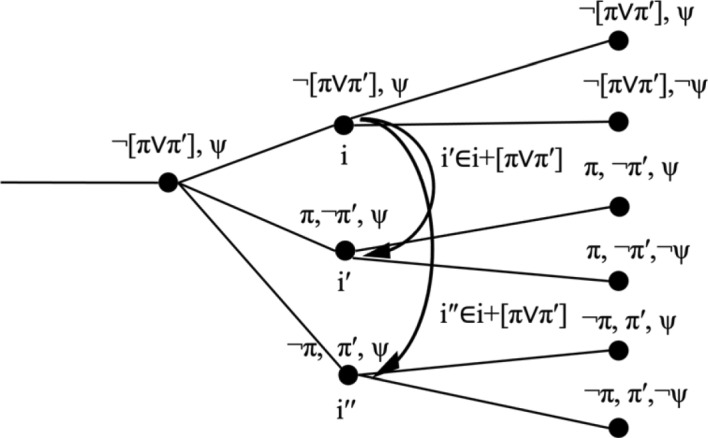

The functional or relational change of indices can be lifted to common grounds, here modelled as context sets, or sets of indices, to define the notion of performative update. For functional index change we have seen this in the definition of performative update in (3), which is equivalent to (14)(a); for relational index change, the relevant formula is (14)(b).

(14) Performative update c + perform(φ)
  $$\begin{aligned}& ({\text{a}}) \,{\text{ Functional index change}} = \{ {\text{i}}^{\prime} \, | \, \exists {\text{i}} \in {\text{c}}\left[ {{\text{i}}^{\prime} = {\text{i}} + \varphi } \right]\} = \{ {\text{i}} + \varphi \, |{\text{ i}} \in {\text{c}}\} \hfill \\& ({\text{b}})\,{\text{ Relational index change}} = \{ {\text{i}}^{\prime} \, | \, \exists {\text{i}} \in {\text{c}}\left[ {{\text{i}}^{\prime} \in {\text{i}} +\varphi} \right]\} = {\bigcup }\{ {\text{i}} + \varphi |{\text{ i}} \in {\text{c}}\} \hfill \\ \end{aligned}$$

Informative update, as defined in (1), is clearly different from performative update, as defined in (14). For informative update c + inform(φ) to be defined, the proposition φ must be a live option in the context c, that is, φ must be true for at least some indices in c. Otherwise, the update would result in the empty set. This is not required for performative update. And indeed, performative utterances like *The meeting is hereby adjourned* are typically expressed when it is common ground that the meeting is *not* adjourned at the current index. Figure (15) visualizes informative and performative update using functional index change for performative update, which is simpler to present graphically.

(15) Informative and performative update of a context setc
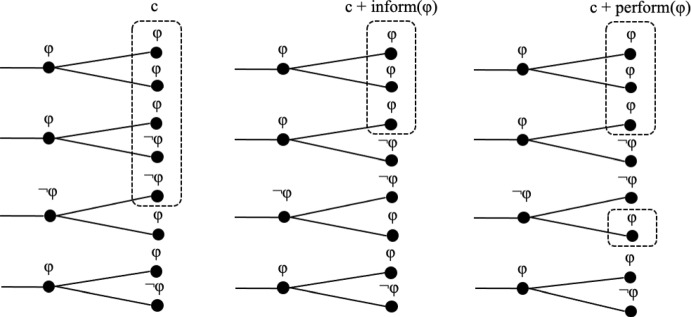

As we see, performative update can introduce indices into a context set that were not in it before. In typical cases of performative updates, the proposition is not true at the indices of a context set. This situation can be illustrated as in (16); note that informative update would result in the empty set.

(16) Informative vs. performative update of a context set
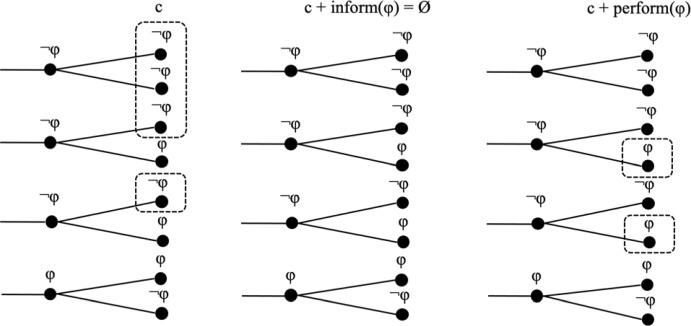

In conclusion: Index change is indeed a coherent notion, either in its functional variety (if we assume that indices do not determine the truth values of all propositions), or in its relational variety. We can model it in frames of branching time, where index change brings about a change from one history to another that does not consume any time.

For the remainder of this paper we will work with relational index change, as this allows for the more familiar notion that indices determine the truth values of all propositions.

## Modeling declarations

With performative updates we have laid the grounds for modeling the speech act of declarations. We take as an example (17), as it would have been uttered by the ZK of the DDR. It is rendered here in English, and we add *hereby* to make it clear that we focus on the reading of this sentence as declaration, not as an assertion.^7^

(17) *Citizens can (hereby) apply for travel abroad.*

We can analyze the declaration (17) uttered at a context set c as the performative update of c with the proposition expressed by the declarative clause it is based on:

(18) $$\begin{aligned} & {\text{c}} + {\text{perform}}\left(\lambda {\text{i}}\left[ {\text{citizens can apply for travel abroad at i}} \right]\right) \end{aligned}$$
  $$\begin{aligned}= \left\{ {\text{i}}^{\prime} \, | \, \exists {\text{i}} \in {\text{c}}\left[ {{\text{i}}^{\prime} \in {\text{i}}} + \lambda {\text{i}}^{\prime \prime}\left[{\text{citizens can apply for travel abroad at }}i^{\prime \prime} \right]\right]\right\}.\end{aligned}$$

How can this interpretation be derived in a compositional way? In linguistics, the syntactic constituent that denotes a proposition is usually identified as Tense Phrase, or TP. We assume that (17) contains such a TP, but also an additional operator that creates the performative update. This operator is not expressed by *hereby*, as this adverbial can be omitted. We rather assume a larger category, an Act Phrase (ActP) with an operator, here denoted by ⋅ in allusion to the sentence-final period in orthography.^8^ This leads to (19) as a simplified representation of the syntactic structure.^9^

(19) $$\left[{\phantom{i}}_{{{\text{ActP}}}} \cdot \left[{\phantom{i}}_{{{\text{TP}}}} citizens \, can \, apply \, for \, travel \, abroad \right]\,\right]$$

The operator ⋅ is interpreted as taking a proposition and yielding a performative update of a context set with that proposition. Assuming that ⟦ α⟧^s,a^ is a function that gives us the meaning of an expression α, where s and a are speaker and addressee, we get the interpretation as in (20), which is put to use in (21):

(20) $${\llbracket {\cdot} \rrbracket} ^{{{\text{s}},{\text{a}}}} = \lambda {\text{p}}\lambda {\text{c}}\{ {\text{i}}^{\prime} \, | \, \exists {\text{i}}\, \in \,{\text{c}}\left[ {{\text{i}}^{\prime} \, \in \,{\text{i}}\, + \,{\text{p}}} \right]\}$$
(21) $$\begin{aligned} & {\llbracket\left[ {{\phantom{i}}_{{{\text{ActP}}}} \cdot \, } \right[{\phantom{i}}_{{{\text{TP}}}} citizens \, can \, apply \, for \, travel \, abroad]]} \rrbracket^{{{\text{s}},{\text{a}}}} \hfill \\ \end{aligned}$$
  $$\begin{aligned} & =  {\llbracket {\cdot} \rrbracket} ^{{{\text{s}},{\text{a}}}} \left( {\llbracket {\left[ {{\phantom{i}}_{{{\text{TP}}}} citizens \, can \, apply \, for \, travel \, abroad} \right]} \rrbracket^{{{\text{s}},{\text{a}}}} } \right) \hfill \\ \end{aligned}$$
  $$\begin{aligned} & = \lambda {\text{c}}\{ {\text{i}}^{\prime} \, | \, \exists {\text{i}} \in {\text{c}}\left[{\text{i}}^{\prime} \in {\text{i}} + \lambda {\text{i}}^{\prime \prime} \left[ {{\text{citizens can apply for travel abroad at }}i^{\prime \prime} } \right]\right]\} . \hfill \\ \end{aligned}$$

The interpretation in (21) does not represent the preconditions that make declarations possible. Searle (1989) famously remarked that performatives like *I hereby fry this egg* would not work, as such physical changes cannot come about by declarations. Also, the index change in (21) can only be enacted by a speaker, like the chairperson of the ZK, that has authority to do so. Such felicity conditions can be represented as presuppositions of updates like (21), which can be modeled by restrictions for the update function, an option already suggested by Szabolcsi (1982). A very general way to express these preconditions is to replace the variable p in the body of (20) by (22).

(22) $$\lambda {\text{i}}^{\prime \prime} .{\text{ s is authorized in i to change i minimally to an }}i^{\prime \prime \prime }{\text{ so that p}}\left( {{\text{i}}^{\prime \prime \prime }} \right). \, [{\text{p}}\left( {{\text{i}}^{\prime \prime} } \right)]$$

This results in a partial propositional function that is only defined for those indices for which the speaker s has the authority to perform the change. The precondition can be extended to include other conditions for performative utterances, like the existence of special institutions and conventions.

Declarations like (17) do not refer to the speaker and do not name the nature of the performative act itself. They are *implicit* performatives (cf. Austin, 1962). In many cases, find that the performative act named explicitly, with 1st person subjects, as in the following examples of *explicit* performatives^10^:

(23) $$\begin{aligned} &  \llbracket{\left[ {{\phantom{i}}_{{{\text{ActP}}}} \cdot \, } \right[{\phantom{i}}_{{{\text{TP}}}} I \, congratulate \, you]]} \rrbracket ^{{{\text{s}},{\text{a}}}} \hfill \\ \end{aligned}$$
  $$\begin{aligned} & = \lambda {\text{c}}\{ {\text{i}}^{\prime} \, | \, \exists {\text{i}} \in {\text{c}}\left[ {{\text{i}}^{\prime} \in {\text{i}} + \lambda {\text{i}}^{\prime \prime} \left[ {{\text{s congratulates a at }}i^{\prime \prime} } \right]} \right]\} . \hfill \\ \end{aligned}$$
(24) $$\begin{aligned} & \llbracket {\left[ {{\phantom{i}}_{{{\text{ActP}}}} \cdot \, } \right[{\phantom{i}}_{{{\text{TP}}}} I \, swear\left[ {{\phantom{i}}_{{{\text{CP}}}} that \, I \, did \, not \, take \, your \, money} \right]]]} \rrbracket^{{{\text{s}},{\text{a}}}} \hfill \\ \end{aligned}$$
  $$\begin{aligned} & = \lambda {\text{c}}\{ {\text{i}}^{\prime} | \, \exists {\text{i}} \in {\text{c}}\left[ {{\text{i}}^{\prime} \in {\text{i}} + \lambda {\text{i}}^{\prime \prime} \left[ {{\text{s swears at }}i^{\prime \prime} {\text{ that s did not take a}}{\text{'}} {\text{s money}}} \right]} \right]\} . \hfill \\ \end{aligned}$$
(25) $$\begin{aligned} & \left[\left[ {\left[ {{\phantom{i}}_{{{\text{ActP}}}} \cdot \, } \right[{\phantom{i}}_{{{\text{TP}}}} I \, promise\left[ {_{{{\text{VPinf}}}} to \, help \, you} \right]]} \right]\right]^{{{\text{s}},{\text{a}}}} \hfill \\ \end{aligned}$$
  $$\begin{aligned} & = \lambda {\text{c}}\{ {\text{i}}^{\prime} \, | \, \exists {\text{i}}\, \in \,{\text{c}}\left[ {{\text{i}}^{\prime} \in {\text{i}} + \lambda {\text{i}}^{\prime \prime} \left[ {{\text{s promises at }}i^{\prime \prime} {\text{ that s will help a}}} \right]} \right]\} . \hfill \\ \end{aligned}$$

In this way, explicit performatives can be subsumed under the more general category of declarations, as suggested in Searle and Vanderveken (1985) and Searle (1989), as well as in Recanati (1987).

The predicates of explicit performatives can generally be used descriptively as well, as in the assertion *Mary congratulated Bill* (cf. Bierwisch, 1980; Recanati, 1987); we will discuss such descriptive uses in the next section.^11^ The current proposal differs from Szabolcsi (1982), who was compelled to assume different semantic types for the descriptive and the performative use of verbs like *promise*. Such type doubling is not necessary in the current proposal, as the operator ⋅ takes a regular proposition and changes it into a performative update. This simplifies the derivation of meanings, and answers a critique of Bach and Harnish (1992) that we should not assume lexical ambiguity between the descriptive and the performative use of such verbs.

## The tense of declarations

In English, declarations are generally rendered in the present tense; for episodic verbs, the simple present is used (*I congratulate you*), not the progressive (*I am congratulating you*). This seems to be a natural choice: In (23), the congratulation happens immediately at the context index i. The progressive, according to standard analyses, would indicate that the congratulation is already ongoing at the index i, and that the final goal of the congratulation might not even be reached (cf. Dowty, 1977).

Interestingly, Szabolcsi (1982) assumed a present perfect interpretation of the proposition in performatives. For *I congratulate you*, Szabolcsi proposes a performative update with the proposition that can be expressed by *I have congratulated you*, that is, λi″∃i‴[i‴ ≤ i″ ∧ s congratulates a in i‴]. That is, the moment of congratulation can be situated before the index i″. But if the index of evaluation i′ would follow the time of the congratulation i‴, then the proposition would be true at i′ already. Hence the current interpretation and Szabolcsi’s present perfect version amount to the same.

Here I do not propose a present perfect interpretation, as explicit performatives are not expressed by this aspect in English. For example, one cannot congratulate someone by saying *I have congratulated you.* But we should not take English as the sole guide of the analysis of performative utterances. Koschmieder (1930), who coined the term “Koinzidenz” for what later became known as explicit performatives, already was aware that languages differ in expressing the coincidence of uttering a sentence and changing the world. In a more recent overview, Fortuin (2019) observes that languages tend to use unmarked verbal forms for explicit performatives. While English uses the simple present, many languages use the present imperfective. This form is motivated by the fact that the performative act happens at the moment of utterance (cf. also Verschueren, 1995). However, present progressives, a subcategory of imperfectives, are avoided (cf. also Bybee & Dahl, 1989).^12^ This is because the progressives refers to protracted events that have an interval as a run time (cf. e.g. Bennett & Partee, 1972 and much subsequent work). The performative update, as modelled here, happens at an instance, and therefore cannot be expressed by the progressive. In terms of Vendler (1957), it is an “achievement”.

Interestingly, Szabolci’s present perfect analysis of explicit performatives is motivated by a number of other languages. Fortuin (2019) points out that there are languages that use perfects, perfectives or even preterites to express explicit performatives. Examples are ancient and modern Afroasiatic languages like Old Egyptian, Akkadian, Ethiopic, Ugaritic, Biblical Hebrew and Arabic (cf. Loesow 2005; Sanders, 2004), but also Ancient Greek and Latin (cf. Bary, 2012; Höfler, 2020), as well as Old Russian and Modern Slovenian^13^ (cf. Dekker, 2018; Močnik, 2015; Škrabec, 1903). In Syriac, explicit performatives are expressed by past participles, thus avoiding a reference to the performer (e.g., instead of “I baptize you” a form corresponding to “You are baptized” is employed, cf. Rogland, 2001). This is not only a past tense form but runs against otherwise observed tendency that explicit performatives have first person subjects (Austin, 1962).

The current proposal is compatible with both present and perfect or perfective clauses if we assume that perfects and perfectives are represented in a way that can include the index at which the tenseless proposition would be interpreted, i.e. if these propositions are of the form λi∃i′[i′ ≤ i ∧ φ(i′)]. But past tense propositions, which are represented by λi∃i′[i′ < i ∧ φ(i′)], should clearly be incompatible with explicit performatives. Indeed, Fortuin (2019) states that perfects and perfectives that otherwise are restricted to past time reference are not suitable for performatives. However, sometimes past tense forms do occur in performatives, such as the preterite in Akkadian (cf. Loesow 2005), the past aorist in Ancient Greek (cf. Bary, 2012) and past tense perfectives in Old Russian (Dekker, 2018). Dekker explains such uses as an effect of writing down an orally performed act, something like epistolary past tense: The oral “I promise to give you ten oxen” would be recorded in writing as “I have promised to give you ten oxen”, hence technically it would be a *report* of a performative. However, this explanation does not apply to the case of Ancient Greek, as the performative use of the past aorist is recorded in direct speech of characters in tragedies. Bary (2012) explains the use of the aorist as a marker of punctual or complete events that may apply to the index of evaluation, as there is no present aorist in the language.

Ancient Greek also allows future tense in performatives, cf. Christensen (2010), and future tense performatives are reported for other languages like Bulgarian, Tibetan, and Tamil as well. A future-tense proposition in a branching-time model is a modal proposition that states that the core proposition becomes true in all histories extending from the current index. We can represent this as λi∀i′[i < i′ → ∃i″[[i < i″ < i′ ∨ i′ ≤ i″] ∧ φ(i′)]] – for all indices i′ after i, the proposition φ either became true between i and i′ or will become true at or after i′. Changing an index i with this futurate proposition results in an i′ for which φ is guaranteed to hold for all future developments of i. Future performatives sometimes are considered more polite than present-tense performatives, which can be attributed t the fact that the core proposition is not enforced on the changed index directly.^14^

We will return to the topic of performative utterances below, in particular in our discussion of performative marker *hereby* in Sect. 9. But we will first turn to assertions, which, as we will argue, have in addition to their informative part also a performative component.

## Modeling assertions

Austin (1962) set out by contrasting constative and performative speech acts, but eventually realized that there are performative aspects in constative speech acts as well: “Surely to state is every bit as much to perform an illocutionary act as, say, to warn or to pronounce” (p. 133).

Indeed, modelling assertions by simple informative updates as in (1) is insufficient. When asserting a sentence, a speaker does not just restrict an input context set by brute force. Rather, reasons must be provided for the addressee to accept this update, and whether the update succeeds depends on the reactions of the addressee. This has been noticed informally in work on dynamic semantics, such as Stalnaker (1978, 2002), and it was integrated into formal approaches that take pragmatic reasoning behind the update into account, such as Farkas and Bruce (2009) and Lauer (2013).

There are various proposals concerning the nature of these reasons that a speaker provides for the addressee to take a proposition as common ground, like a conversational maxim of quality (Grice, 1975), a convention of truthfulness and trust (Lewis, 1975), the expression of a belief (Bach & Harnish, 1979), or the expression of a wish that the addressee should come to believe the proposition as well (Truckenbrodt, 2006). Here I will follow the commitment theory of assertion which goes back to Charles Sanders Peirce (cf. Tuzet, 2006) and which was developed in much recent work (cf. Brandom, 1983, Condoravdi & Lauer, 2011, Geurts, 2019, and Shapiro, 2020 for an overview). More specifically, the Peircian view assumes that with asserting a proposition the speaker gives a *guarantee* for the truth of the proposition – the speaker *vouches for* the truth of the proposition. This is essentially a *social* notion: The speaker undergoes a risk of losing reputation of trustworthiness in case the proposition turns out to be false. These social consequences are the reason why the addressee takes the speaker serious, and why the addressee will come to assume the proposition as well.

The public commitment for the truth of a proposition is the performative part of an assertion. It can be expressed by a proposition as well, the proposition that the speaker guarantees for the truth of the asserted proposition. We express this here with Frege’s judgement stroke (even though Frege, in contrast to Peirce, did not appeal to the social dimension of assertions):

(26) $$\lambda {\text{i}}\left[ {{\text{x}} { \vdash }_{{\text{i}}} \varphi } \right]\, = \,\lambda {\text{i}}[{\text{x guarantees in i that }}\varphi {\text{ is true in i}}]$$

Assertions are treated as performative updates with a truth commitment by the speaker. We get the following interpretation of the assertive interpretation of our core example:

(27) $$\begin{aligned} & {\text{S}}:Citizens \, can \, apply \, for \, travel \, abroad \hfill \\ \end{aligned}$$
  $$\begin{aligned} \lambda {\text{c}}\{ {\text{i}}^{\prime} \, | \, \exists {\text{i}} \in {\text{c}}\left[ {{\text{i}}^{\prime} \in {\text{i}} + \lambda {\text{i}}^{\prime \prime} } \right[{\text{S\;guarantees in }}i^{\prime \prime} \hfill \\ \end{aligned}$$
  $$\begin{aligned} & {\text{ that `citizens can apply for travel abroad}}{\text{'}} {\text{ is true in }}i^{\prime \prime} ]]\} . \hfill \\ \end{aligned}$$

The input context set c is performatively changed so that the speaker S guarantees for the truth of the proposition, that citizens can apply for travel abroad.

How do we get to this interpretation? The performative update is mediated by the operator ⋅ as before, but we have to assume an additional operator that introduces the speaker’s public commitment. An operator of this type was assumed in the performative hypothesis of assertions (Lewis, 1970; Ross, 1970), a hypothesis that was critically discussed but is recently revived in various syntactic work (cf. e.g. Hengeveld, 1989; Speas & Tenny, 2003; Wiltschko, 2021). Following Miyagawa (2022) and Krifka (2023), we will assume a *commitment phrase* ComP with a syntactic head that introduces an operator for which we will also use the symbol ⊢. We then have the following syntactic structure for the assertion^15^:

(28) $$[{\phantom{i}}_{{{\text{ActP}}}} \cdot \,  [{\phantom{i}}_{{{\text{ComP}}}} { \vdash } \, \left[ {{\phantom{i}}_{{{\text{TP}}}} citizens \, can \, apply \, for \, travel \, abroad} \right]]].$$

With the interpretation of the commitment operator as in (29), we get the compositional interpretation as in (30).

(29) $$\llbracket { \vdash } \rrbracket^{{{\text{s}},{\text{a}}}} = \lambda {\text{p}}\lambda {\text{i}}[{\text{s}} { \vdash }_{{\text{i}}} {\text{p}}].$$
(30) $$\begin{aligned} &  \llbracket {\left[ {{\phantom{i}}_{{{\text{ActP}}}} \cdot \, } \right[{\phantom{i}}_{{{\text{ComP}}}} { \vdash } \, \left[ {{\phantom{i}}_{{{\text{TP}}}} citizens \, can \, apply \, for \, travel \, abroad} \right]]]} \rrbracket^{{{\text{s}},{\text{a}}}} \hfill \\ \end{aligned}$$
  $$\begin{aligned} & = \llbracket \cdot \rrbracket^{{{\text{s}},{\text{a}}}} ( \llbracket { \vdash } \rrbracket^{{{\text{s}},{\text{a}}}} \left( {\llbracket {\left[ {{\phantom{i}}_{{{\text{TP}}}} citizens \, can \, apply \, for \, travel \, abroad} \right]} \rrbracket^{{{\text{s}},{\text{a}}}} } \right)) \hfill \\ \end{aligned}$$
  $$\begin{aligned} &= \lambda {\text{c }}\left\{ {\text{i}}^{\prime} \, | \, \exists {\text{i}}\, \in \,{\text{c}}\left[ {{\text{i}}^{\prime} \in {\text{i}} + \lambda {\text{i}}^{\prime \prime} } \right[{\text{s}} { \vdash }_{{{\text{i}}^{\prime \prime} }} \lambda {\text{i}}^{\prime \prime \prime }\left.\left.\left.\left[{\text{citizens can apply for travel abroad in }}i^{\prime \prime \prime }\right]\right]\right]\right\}\right. . \hfill \\ \end{aligned}$$

Let us consider the press conference on November 9, 1989, whose context set we represent by c_0_.

(31) $$\begin{aligned} & {\text{c}}_{{\text{o}}} + {\text{Schabowski}}:Citizens \, can \, apply \, for \, travel \, abroad \hfill \\ \end{aligned}$$
  $$\begin{aligned} & = {\text{c}}_{{\text{o}}} + {\text{perform}}(\lambda {\text{i}}[{\text{Sch}}\;{ \vdash }_{{\text{i}}} {\text{citizens can apply for travel abroad}}{\text{'}} ) \hfill \\ \end{aligned}$$
  $$\begin{aligned} & = \left\{ {{\text{i}}^{\prime} | \, \exists {\text{i}} \in {\text{c}}_{{\text{o}}} \left[ {{\text{i}}^{\prime} \!\in\! {\text{i}} \!+\! \lambda {\text{i}}\left[ {{\text{Sch }}{ \vdash }_{{\text{i}}} {\text{citizens can apply for travel abroad}}{\text{'}} } \right]} \right]} \right\} \!=\! {\text{c}}_{1} \hfill \\ \end{aligned}$$

This is a performative update. In the indices of the input context set c_0_, Schabowski was not guaranteeing for the truth of the proposition ‘citizens can apply for travel abroad’; in the indices of the output context set c_1_, he is.

At this point, the proposition ‘citizens can apply for travel abroad’ is not yet part of c_1_. But the public commitment of Schabowski, who holds the authority of the chief information officer of the ZK of the SED, is a good reason for the addressees (the journalists, and subsequently, the citizens of East Germany) to believe the truth of the proposition. This second update is purely informative:

(32) $$\begin{aligned} & {\text{c}}_{{1}} + {\text{inform}}({\text{`citizens can apply for travel abroad}}{\text{'}} ) \hfill \\ \end{aligned}$$
  $$\begin{aligned} &\quad = \{ {\text{i}} \in {\text{c}}_{{1}} \, |{\text{ `citizens can apply for travel abroad}}{\text{'}} \left( {\text{i}} \right)\} \hfill \\ \end{aligned}$$

The informative update is the primary purpose of the assertion; the performative update is just a tool to achieve that goal. We can identify the performative update as the *illocutionary* act of an assertion and the informative update as its primary *perlocutionary* act: The speaker wants the addressee to accept the proposition into the common ground.

There are various ways how the informative update can be dealt with in the semantic interpretation of assertions. One option is to leave it up to pragmatics, as a mere *conversational implicature*: With guaranteeing the truth of a proposition, the speaker undergoes a social risk; a rational reason for undertaking this risk is that the speaker wants to give a reason for the addressee to accept this proposition. However, there seems to be more to an assertion than just that. Conversational implicatures can be cancelled (cf. Grice, 1975), but this appears to be difficult with the informative part of assertions: *It is raining but I don’t care whether you believe it* is close to a pragmatic paradox.^16^ Also, if a speaker says *I hereby vouch for the truth of φ* this does not quite amount to that the speaker *told* the addressee that φ, because telling someone that φ typically includes at least some effort of the speaker to convince the addressee that φ is true.

This leads to a semantic representation of assertions that includes the intended informative update of the asserted proposition, in addition to the performative update of vouching for the truth of that proposition. The simplest option is to model assertions as a dynamic conjunction, i.e. functional composition, of performative update followed by informative update. This is expressed in (33) with the operator “;”.

(33) $$\begin{aligned} & \lambda {\text{c}}\{ {\text{i}}^{\prime} \left| { \, \exists {\text{i}}\, \in \,{\text{c}}\left[ {{\text{i}}^{\prime} \, \in \,{\text{i}}\, + \,\lambda {\text{i}}^{\prime \prime} \left[{\text{s}} { \vdash }_{{{\text{i}}^{\prime \prime}}} \varphi\right] } \right]\} ; \, \lambda {\text{c}}\{ {\text{i}}^{\prime \prime \prime }\, \in \,{\text{c}} } \right| \varphi \left( {{\text{i}}^{\prime \prime \prime }} \right)\} \hfill \\ \end{aligned}$$
  $$\begin{aligned} & \quad = \lambda {\text{c}}\left\{ {\text{i}}^{\prime \prime \prime }\, \in \,\left\{ {\text{i}}^{\prime} \right. \left| { \, \exists {\text{i}}\, \in \,{\text{c}}\left[ \left.{{\text{i}}^{\prime} \, \in \,{\text{i}}\, + \,\lambda {\text{i}}^{\prime \prime} \left[ {\text{s}} { \vdash }_{{{\text{i}}^{\prime \prime} }} \varphi \right]} \right] \right\} } \right| \varphi \left( {{\text{i}}^{\prime \prime \prime }} \right)\right\} \hfill \\ \end{aligned}$$
  $$\begin{aligned} &\quad = \lambda {\text{c}}\left\{ {\text{i}}^{\prime \prime \prime }| \exists {\text{i}}\, \in \,{\text{c}}\left[ {{\text{i}}^{\prime \prime \prime }\, \in \,{\text{i}}\, + \,\lambda {\text{i}}^{\prime \prime} \left[ {\text{s}} { \vdash }_{{{\text{i}}^{\prime \prime} }}. \varphi \right]}\right] { \, \wedge \, \varphi \left( {{\text{i}}^{\prime \prime \prime }} \right)} \right\} . \hfill \\ \end{aligned}$$

However, this does not capture the fact that the acceptance of the informative update depends on the reaction of the addressee. In case the addressee replies with *No*, the proposition φ itself will not become part of the common ground. This can be modeled in various ways. There are theories that propose automata-theoretic devices such as Merin (1994) and Farkas and Bruce (2010). Krifka (2015) proposes a framework which allows for the retraction of the informative update when the addressee rejects it. Krifka (2022b) develops a representation within the framework of Commitment Spaces, which model the possible continuations of context sets; after committing to the truth of a proposition, the speaker offers a disjunction of the informative update or requests some reaction of the addressee that blocks that informative update.

In contrast to the informative update, the performative update, by which the speaker guarantees for the truth of a proposition, cannot be rejected by the addressee. It is only in the authority of the speaker to remove this guarantee; what the addressee can do is to ask the speaker for its removal, as in *Don’t say that!* or *Take this back!* These are also possible reactions for explicit performatives, and declarations in general, which also may be removed by the speaker as the authority that introduced them in the first place. In certain cases, the addressee may also question the authority of the speaker, and in this way the performative update will be rejected.

One further point is worth mentioning. In our proposal, we have assumed a structural ambiguity between the declaration and the assertion of sentences like *citizens can apply for travel abroad*, cf. (19) vs. (28). Declarations are not treated as assertions that guarantee their own truth, as in Bach and Harnish (1979) and Condoravdi and Lauer (2011), but as having a different syntactic representation and semantic interpretation (cf. also Jary, 2007; Reimer, 1995; Searle, 1989). According to the current proposal, declarations have a simpler syntactic structure than assertions, as they lack a commitment phrase, and consequently do not involve a truth commitment by the speaker to a proposition that is used to make the addressee accept the truth of that proposition. Rather, with a declaration made under the right circumstances, a speaker can make the proposition true by brute force.

One linguistic piece of evidence that declarations are not based on assertions is that they do not allow for sentence adverbials like *truly*, *certainly* and *apparently* that address the commitment of the assertion (cf. Krifka, 2023). Furthermore, assertions come with the sister speech act of questions, which can be seen as requests for assertions by the addressee and which can be modeled due to the presence of a Commitment Phrase (cf. Krifka, 2022a). Questions cannot be used to request declarations; e.g. in the sequence A: *Is the border open? –* B: *Yes*, B’s answer can only be understood as an information about an existing state of affairs, not as a performative change of that state of affairs.

## Other speech acts

We have seen that declarations and assertions both bring about a performative change, in the former case an index change so that the core proposition becomes true, and in the latter case a guarantee that the asserted proposition is true. In fact, all speech acts bring about a performative change. I would like to illustrate this with commissives, exclamatives, optatives, definitions, imperatives and a speech act type I tentatively call “proxitive”.

Commissives, like many other speech acts, can be expressed by explicit performatives such as *I promise to help you*, as analyzed in (25). With this, the speaker changes the indices of the context set so that they now contain an obligation for the speaker to act in a specified way. But promises can be expressed with future clauses as well, such as *I will help you*. Such sentences can be interpreted as assertions or as declarations, cf. (34)(a), and (b):

(34) $$\begin{aligned} & {\text{a}}.\,\llbracket { \left[ {{\phantom{i}}_{{{\text{ActP}}}} \cdot \, } \right[{\phantom{i}}_{{{\text{ComP}}}} { \vdash } \, \left[ {{\phantom{i}}_{{{\text{TP}}}} I \, will \, help \, you} \right]]]} \rrbracket^{{{\text{s}},{\text{a}}}} \hfill \\ \end{aligned}$$
  $$\begin{aligned} & \quad = \lambda {\text{c}}\left\{ {\text{i}}^{\prime} \, | \, \exists {\text{i}}\, \in \,{\text{c}}\left[ {{\text{i}}^{\prime} \, \in \,{\text{i}}\, + \,\lambda {\text{i}}\left[ {{\text{s}} { \vdash }_{{\text{i}}} \lambda {\text{i}}^{\prime \prime} } \right[{\text{s will help a in }}i^{\prime \prime} } \right]\right]\} \, ( + \,{\text{informative update}}). \hfill \\& {\text{b}}.\, \llbracket {\left[ {{\phantom{i}}_{{{\text{ActP}}}} \cdot \, }\right[{\phantom{i}}{{{\text{TP}}}} I \, will \, help \, you]]]} \rrbracket ^{{{\text{s}},{\text{a}}}} \\ &\quad = \,\lambda {\text{c}}\,\{ {\text{i}}^{\prime} | \, \exists {\text{i}}\, \in \,{\text{c}}\left[{\text{i}}^{\prime} \, \in \,{\text{i}}\, + \,\lambda {\text{i}}^{\prime \prime} \left[  {{\text{s will help a in }}i^{\prime \prime} } \right] \right]\} \hfill \\& {\text{where }}\lambda {\text{i}}\left[ {\text{s will help a in i}} \right]\\ &= \,\lambda {\text{i}}\forall {\text{i}}^{\prime} \left[ {{\text{i}}\, < \,{\text{i}}^{\prime} \, \to \,\exists {\text{i}}^{\prime \prime} } \right[{\text{i}}\, < \,{\text{i}}^{\prime \prime} \, \le \,{\text{i}}^{\prime} \, \vee {\text{ i}}^\prime < {\text{i}}^{\prime \prime} \, \wedge \, \left. \left. \left[ {{\text{s helps a in }}i^{\prime \prime} } \right] \right] \right]. \hfill \\ \end{aligned}$$

With the assertion, the speaker commits to the truth of the proposition that he or she will help the addressee; with the explicit performative, the speaker simply makes this proposition true, for the indices of the context set. The future proposition is the same in both cases; it is true for all indices i that are followed by a history in which the core proposition becomes true at one point.^17^ The two interpretations can be distinguished insofar as the first allows for assertion-specific modifiers such as epistemic modifiers like *certainly*.

Turning to directives, we observe that they also can be expressed explicitly, as in *I ask you to help me*, but there is also a grammaticalized form, the imperative. There are several proposals for the modeling of imperatives, e.g. by a conventionalized form of the explicit performative *I (hereby) command that…* (cf. Lewis, 1970), by a separate component of the common ground, so-called to-do lists (Portner, 2004) or action plans (Mastop 2011), by performative deontic modals similar to *You must help me* (Kaufmann, 2012), by action-denoting expressions (Barker, 2012; Segerberg, 1990), or as imposing a preference relation on the propositions in the common ground (Condoravdi & Lauer, 2012; Starr, 2020). These proposals have in common that directives have a performative, not an informative meaning; they change the world by creating an obligation to the addressee that did not exist before. The analysis of performatives developed here is compatible with and provides the theoretical underpinning for different theories of imperatives. For example, it can render the meaning of imperatives like *Help me!* as performative update with an obligation for the addressee to help the speaker:

(35) $$\begin{aligned} & \llbracket {\left[ {{\phantom{i}}_{{{\text{ActP}}}} Help \, me!} \right]} \rrbracket^{{{\text{s}},{\text{a}}}}{=}\lambda {\text{c}}\left\{ {{\text{i}}^{\prime } \, | \, \exists {\text{i}} \in {\text{c}} \left[{\text{i}}^{\prime } \in {\text{i}}{+}\lambda {\text{i}}^{\prime \prime} \forall {\text{i}}^{\prime \prime \prime } \in {\text{OBL}}({\text{i}}^{\prime \prime } )({\text{a}})\left[ {{\text{a will help s in i}}^{\prime \prime \prime } } \right]\right]} \right\}, \hfill \\ \end{aligned}$$
  $$\begin{aligned} & {\text{where OBL}}\left( {\text{i}} \right)\left( {\text{a}} \right) = {\text{the set of indices that are compatible with a}}{\text{'}} {\text{s obligations in i}}. \hfill \\ \end{aligned}$$

Alternatively, imperatives can be analyzed as proposals to change the context set with the future action of the addressee as in (36). In doing this, it becomes part of the common ground that the addressee will help the speaker. This does not mean that the addressee will help for sure – reality, after all, can develop in ways that differ from the common ground.

(36) $$\llbracket {\left[ {{\phantom{i}}_{{{\text{ActP}}}} Help \, me!} \right]} \rrbracket^{{{\text{s}},{\text{a}}}} = \lambda {\text{c}}\left\{ { {\text{i}}^{\prime} \, | \, \exists {\text{i}}\left[ {{\text{i}}^{\prime} {\epsilon}{\text{ i}}\, + \,\lambda {\text{i}}^{\prime \prime} \left[ {{\text{a will help s in }}i^{\prime \prime} } \right]} \right]} \right\}.$$

Representations as in (35) and (36) would have to be complemented by ways for the addressee to express non-compliance, e.g. by disjunctions as proposed in Krifka (2022a, 2022b). Also, notice that directives can be expressed by deontic modals, as in *You must help me!* and by future-oriented propositions, as in *You will help me!*, which can be seen as declarations. This raises the issue in which way true imperatives such as *Help me!* are different.

We turn to exclamatives such as *Wow! The borders are open!* and other types like *Is she fast!* and *How fast she is!* (cf. Rett, 2011). Exclamatives obviously communicate emotional attitudes, or epistemic attitudes of surprise, about a true proposition, e.g., that the borders are actually open, or that Mary is fast to the degree she actually is. But with exclamatives, speakers do not vouch for the truth of propositions concerning their emotional or epistemic attitude, as in *I am surprised that the borders are open.* Rather, speakers *display* this attitude. Such displays are performative: The speaker changes the indices of the context set by expressing this attitude.

Another type of speech acts that lend themselves to a performative analysis are optatives (cf. Grosz, 2011). An optative like *If only the borders were open!* differs from the assertion of wishes like *I wish that the borders were open* in similar ways as exclamatives, as displays of surprise, differ from assertions about surprise. Both optatives and the assertion of wishes involve the relevant proposition ‘speaker wishes/prefers that φ’, but with assertions, the speaker guarantees that this proposition is true, whereas with wishes, the speaker induces an index change that makes this proposition true.

There are also speech acts that impose a proposition as a fact, at least for the time being. For example, in mathematical texts a statement like *Let x be a prime number* establishes the proposition ‘x is a prime number’ as true, by virtue of the authority of the speaker. A similar case are definitions that regulate the use of language, as in the case of giving a name to a person or ship, cf. Austin (1962). Such definitional acts are obviously performative, as they enact a change of the social rules how language is used.

As a last type of performative act I would like to mention one that is only recently getting attention. Bücking and Rau (2013) discuss a novel use of non-inflected verb forms in internet chats in German that are often framed by asterisks, such as *grins* ‘smile’ or *unschuldigguck* ‘look innocently’. They are used as proxy for the act of smiling (and the communicative function that this act may have). Emoticons like ***, ***, or acronyms like *lol* ‘laughing out loud’ or *rofl* ‘rolling on the floor laughing’ have a similar function. Such speech acts are clearly performative in nature: the speaker does not *inform* the addressee that there is a certain emotional state but *displays* this state. They can also be modeled by performative updates that change the indices of the common ground. There is no accepted term for such speech acts (the German cases are called “inflective”, as they are formally expressed by the non-inflected root of the verb, but this does not describe their function). I suggest the term “proxitive”, as the linguistic expression or semiotic sign stands proxy for another act.

## The locutionary act

So far we have dealt with illocutionary acts, analyzed as creations of new facts like a change of legal rules in the case of declarations, or a person vouching for the truth of a proposition in the case of assertions. We have also talked about certain perlocutionary acts, like introducing a proposition into the common ground in case of assertions. What about the *locutionary* acts, the third aspect of speech acts that Austin discusses?

There is considerable discussion about what Austin meant by this term (cf. e.g. Kissine, 2008; Sbisà, 2013). Austin understood it as “uttering a sentence with a certain sense and reference” (1962, p. 108). We take it as the production of a linguistic form under a particular structure, with certain prosodic and also gestural features. The locutionary act is an essential cause for the illocutionary act to occur. If Sue wants to congratulate Max, she has to *say* something to this effect, and perhaps perform other actions like shaking his hands, patting his back, or handing over a token of appreciation. That is, an event of a particular type with identifying features has to occur. This event is temporally protracted, different from what we assumed for the illocutionary act. How can this be modeled?

Being temporally extended, locutionary acts cannot become true at instances, like illocutionary acts. Rather, they are events that take time to develop. Hence we switch to an event-semantics framework (Davidson, 1967). Events happen at intervals, which can be considered as convex sets of indices on a branch of development, i.e. I is an interval iff there are indices i, i′ such that i, i′ ∈ I and ∀i″[i ≤ i″ ≤ i′ ↔ i″ ∈ I].^18^ (37) illustrates how a proposition that informs about an extended past event can be represented; the proposition holds for indices iff they are preceded by (all the indices of) an interval I and there is an event e that happens at I and is an event of talking by Sue to Max.

(37) $$\llbracket {\left[ {{\phantom{i}}_{{{\text{TP}}}} Sue \, talked \, to \, Max} \right]} \rrbracket^{{{\text{s}},{\text{a}}}} \, = \,\lambda {\text{i}}\exists {\text{e}}\exists {\text{I}}[{\text{I}}\, < \,{\text{i }} \wedge {\text{ talk}} \text{-} {\text{to}}\left( {\text{I}} \right)\left( {\text{e}} \right)\left( {{\text{max}}} \right)\left( {{\text{sue}}} \right)].$$

In order to describe locutionary acts we have to refer to the wording used by the speaker. Let us introduce a general predicate SAY that takes a linguistic representation as an argument, together with the speaker, the addressee, and an event. Here is a non-performative example that illustrates its use:

(38) $$ \begin{aligned} & \llbracket {\left[ {{\phantom{i}}_{{{\text{TP}}}} Sue \, said \, "I \, congratulate \, you" \, to \, Max} \right]} \rrbracket^{{{\text{s}},{\text{a}}}} \hfill \\ \end{aligned} $$
  $$ \begin{aligned} &\quad = \lambda {\text{i}}\exists {\text{e}}\exists {\text{I}}[{\text{I}}\, < \,{\text{i }} \wedge {\text{ SAY}}\left( {\text{I}} \right)\left( {\text{e}} \right)\left( {\left[ {I \, congratulate \, you} \right]} \right)\left( {{\text{max}}} \right)\left( {{\text{sue}}} \right)]. \hfill \\ \end{aligned} $$

Locutionary acts are performative, in the sense that they change the world. For example, when Sue says *I congratulate you* to Max, perhaps accompanied with a smile, a handshake, or a gift, Sue is doing something in the world, like moving her articulators and generating sound waves. Concentrating on the linguistic part of that, we can represent this performative update as follows:

(39) Sue says to Max:
  $$\begin{aligned} & [{\phantom{i}}_{{{\text{ActP}}}} \cdot \left[ {{\phantom{i}}_{{{\text{TP}}}} I \, congratulate \, you} \right]] \hfill \\&\quad = \lambda {\text{c}}\{ {\text{i}}^{\prime \prime \prime }\, | \, \exists {\text{i}}\, \in \,{\text{c}}\exists {\text{i}}^{\prime} [ {\text{i}}^{\prime} \, \in \,{\text{i}}\, + \,\lambda {\text{i}}^{\prime \prime} \exists {\text{e}}\exists {\text{I}}[{\text{i}}^{\prime \prime} \, = \,{\text{ini}}\left( {\text{I}} \right) \, \wedge {\text{ }}i^{\prime \prime \prime }\, = \,{\text{fin}}({\text{I}}) \hfill \\& \wedge {\text{ SAY}}\left( {\text{I}} \right)\left( {\text{e}} \right)\left( {\left[ {I \, congratulate \, you} \right]} \right)\left( {{\text{max}}} \right)\left( {{\text{sue}}} \right)]]\} . \hfill \\ \end{aligned}$$

This changes the indices i of the input context c first to indices i′ that are minimally different from i insofar they are the initial point of an interval I at which there is an event e, where Sue says to Max, “I congratulate you”. The output indices i‴ are then the final points of these intervals.

The performative update with the locutionary act, which consists in *pronouncing* a linguistic expression, is followed by the performative update with the illocutionary act, which consists of *interpreting* this expression. Figure (40) is an attempt to illustrate this in detail. The input context set c_0_ represents the information that is mutually shared at the point where Sue makes this utterance. When Sue starts to pronounce the first word, she initiates a change of the indices in c_0_, hence this is a performative, not an informative update, with the resulting context set c_1_. As the utterance is directly accessible to the participants, we can generally assume that for every index i in c_0_ there is a unique index that differs from i only insofar as the initial part of the utterance is realized by the speaker, in precisely the way how the speaker realized it. The pronunciation of the first word *I* leads to the context set c_2_, the pronunciation of the second word *congratulate* to c_3_ and the pronunciation of the third word *you* to c_4_. The indicated changes may include other events that are part of the shared attention during the uttering of this sentence.^19^

(40) Update of a context set with a locutionary act and illocutionary act.
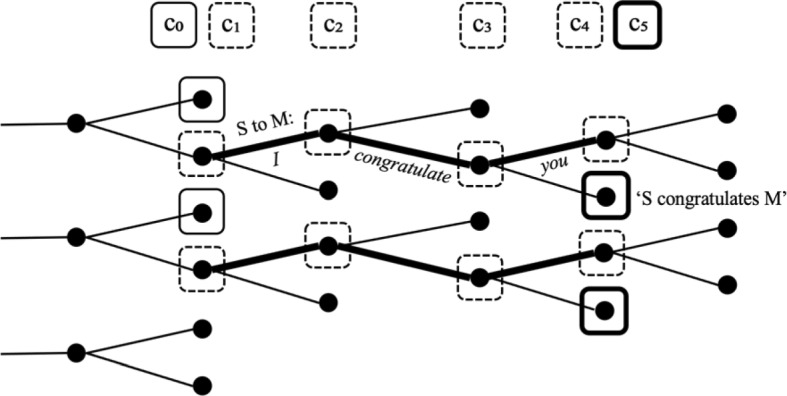

In the last step, after the utterance (the locutionary act) is completed, the illocutionary effect arises: The indices of the context set c_4_ are updated with the proposition ‘Sue congratulates Max’, as a result of the utterance of the sentence. This results in context set c_0_.

We can generalize this proposal as follows. Locutionary acts are based on a linguistic form *α* performed by a speaker s to an addressee a. Let us use double angled brackets for the the *phonological* interpretation, or realization, of a linguistic form:

(41) $$\left\langle\!{\left\langle \alpha \right\rangle}\! \right\rangle^{{{\text{s}},{\text{a}}}} = \lambda {\text{c}}\{ {\text{i}}{} \, | \, \exists {\text{i}}\, \in \,{\text{c}}\exists {\text{i}}^{\prime} [ {\text{i}}^{\prime} \, \in \,{\text{i}}\, + \,\lambda {\text{i}}^{\prime \prime} \exists {\text{e}}\exists {\text{I}}[ {\text{i}}^{\prime \prime} \, = \,{\text{ini}}\left( {\text{I}} \right) \, \wedge {\text{ i}}{}\, = \,{\text{fin}}\left( {\text{I}} \right) \, \wedge {\text{ SAY}}\left( {\text{I}} \right)\left( {\text{e}} \right)\left( \alpha \right)\left( {\text{a}} \right)\left( {\text{s}} \right)]\} .$$

Locutionary acts trigger a performative change of the utterance of some linguistic form *α*. If *α* is of a form that encodes a speech act, the illocutionary effect that the locutionary act of uttering *α* brings about is the application of the *semantic* interpretation of *α* at the output of the phonetic interpretation. That is, the locutionary act is dynamically conjoined, or composed with, the illocutionary act:

(42) $$\left\langle\!{\left\langle \alpha \right\rangle}\! \right\rangle^{{{\text{s}},{\text{a}}}} ; \, \llbracket \alpha \rrbracket^{{{\text{s}},{\text{a}}}} = \lambda {\text{c}}\left[ {\llbracket \alpha \rrbracket^{{{\text{s}},{\text{a}}}} \left( {\left\langle\!{\left\langle \alpha \right\rangle}\! \right\rangle^{{{\text{s}},{\text{a}}}} \left( {\text{c}} \right)} \right)} \right]$$

A complete speech act consists in (at least) a combination of a locutionary act and an illocutionary act. Without the former, the latter would not arise. In this sense, the locutionary act can be considered the “cause” of the illocutionary act. But the causal relation does not have to be rendered explicitly, as there is simply no way that an illocutionary act happens on its own.

The interpretation pattern in (42) suggests that words and constituents are put together in a way that is accessible to the participants in conversation, leading to a successive change of the context set, till they form a constituent α whose meaning is a speech act that can be applied to that context set. In this sense, speech acts are the “minimal units of conversation” (Searle, 1969). These units may be indicated by prosody, as proposed by Truckenbrodt (2015). Also, the procedure may favor placement of explicit speech-act related segmental marking at the end of the constituent that is interpreted as speech act, such as sentence-final particles.

The combination of a locutionary act and the illocutionary act form what would be denoted by accomplishment verbs in the sense of Vendler (1957) and Parsons (1990): The former is an event that takes time and culminates in the latter. Van Lambalgen and Hamm (2005: 49ff.) develop a detailed representation of accomplishments that involves events with a plan for a particular goal to be reached; this is a plausible framework for the description of speech-act verbs. Event-semantic accounts for speech acts have been proposed by a number of authors, e.g. Poesio and Muskens (1997), Brasoveanu and Farkas (2007), Kissine (2008). Eckardt (2012); proposes Davidson’s theory of action to describe the relation between locutionary and illocutionary act.

We have seen that English uses simple present tense for explicit performatives. The instantaneous nature of illocutionary acts prevents the use of the progressive, an aspectual form that requires an event that takes an interval as run time. Hence, sentences like *I am congratulating you* are understood as referring to the locutionary act as an ongoing event. As the illocutionary effect takes place only at the final point of the locutionary act, this sentence does not entail that the illocutionary effect obtains, and hence is less suitable as an explicit performative.

One might ask when exactly the illocutionary effect happens with respect to the locutionary act. For certain performatives, the precise timing of the change can be of importance, and then can be marked by an instantaneous signal. For example, in some types of auctions the auctioneer marks the end of the bidding with the knock of a gavel on the lectern, followed by announcing the winning price and the successful bidder. The formula *Ready, set, go!* is a standard locutionary act that starts a race; runners are allowed to start running at the end of *go*, which is often marked by an additional sharp signal, like a pistol shot, or a particularly short realization of the word *go*. But for most performatives, the precise timing of the illocutionary change is not particularly relevant. But this does not mean that the illocutionary changes themselves are protracted.

## The role of *hereby*

Certain performative utterances can be marked by *hereby*. Eckardt (2012), who develops an event-based theory of explicit performative acts, analyzes this term as referring to the event of the locutionary act in which it occurs (the “ongoing act of information transfer”). Eckardt analyzes explicit performatives as in (43), where *hereby* deictically refers to the very utterance of the sentence, which is classified as a promise.

(43) $$\begin{aligned} & {\text{e}}_{{\text{o}}} :{\text{ Max}}:I\;hereby \, promise \, to \, clean \, the \, kitchen. \hfill \\ \end{aligned}$$
  $$\begin{aligned} & {\text{Interpretation}}: \, \lambda {\text{w}}\left[ {{\text{promise}}({\text{max}},{\text{ e}}_{{\text{o}}} ,{\text{ w}}, \, \lambda {\text{w}}^{\prime} \lambda {\text{e}}} \left[{\text{clean}}( {{\text{max}},{\text{ e}},{\text{ w}}^{\prime} ,{\text{k}}} \right])\right]. \hfill \\ \end{aligned}$$

This account works fine with performatives that label the speech act they express – the act of information transfer e_0_ in (43) is a promise, and hence can fill the event argument position of *promise*. It is not so clear how this applies to examples like *I hereby open the exhibition* (an example mentioned by Eckardt), as *open* is not clearly a verb that can be applied to an act of information transfer. The account is problematic for declarations that are not explicit performatives, as in *The meeting is hereby adjourned* or *All rights are hereby reserved.* This also applies to the account of Močnik (2015).

Furthermore, the analysis gives us a good explanation for the *here* in *hereby*: It is a proximate deictic, in contrast to *there*, and hence is plausible as a reference to the ongoing speech act. But it does not explain the *by* part, which should play the same role as in *thereby*. The lexical meaning of this part expresses causality; the Oxford English Dictionary gives as meaning of *hereby* “By, through, or from this fact or circumstance; as a result of this; by this means”. The German equivalent *hiermit* derives from an instrumental meaning, and is often used in a way that does not relate to speech acts but to instruments.

There is also a problem with performatives without *hereby*. Eckardt argues for an analysis in terms of existential binding of the event variable in a way that allows it to be identified with the ongoing act of information transfer. Hence there is no systematic distinction between the reportative and the performative interpretation. This might be fine for English, where the simple present tense with first-person subject sentences does not leave other good choices to anchor the event variable than the ongoing act of information transfer. It is not so plausible for German, were present tense sentences easily receive a future interpretation. Still, a sentence like *Ich verspreche, die Küche aufzuräumen* (the translation of (43) without *hereby*) receives a performative analysis, whereas *Ich gehe zum Einkaufen* (lit. ‘I go shopping’) easily receives a future-oriented reportative interpretation.

Recall that in our analysis, the illocutionary act of a sentence like (43) induces a performative update with the result that the proposition ‘Max promises to clean the kitchen’ becomes true, with the effect that Max has the obligation to clean the kitchen. This analysis applies to declarations like *The meeting is (hereby) adjourned* as well, so it has greater generality compared to Eckardt’s proposal.

The question arises what *here-* in *hereby* refers to. The obvious candidate is the utterance event, the argument of the SAY predicate, that is introduced by the locutionary act, cf. (41). This is present in both explicit performatives and other declarations. In a more comprehensive theory, we would have to assume that the utterance event introduces a discourse referent that can be picked up by a deictic term, just as other entities that are present in the situation of utterance (cf. Buch, 2021, 2023). When considering actual uses of *hereby*, we find that it is often referring to a particular concomitant event, not the pure locutionary act, such as a signature under a contract, or a gesture like a handshake, and hence occurs in more formal contexts. In contrast, German *hiermit* is used more liberally.^20^

There is also the question what the causative meaning expressed by *by* refers to. One obvious candidate is that the locutionary act is the cause by which the illocutionary act comes about, in the representation of (42). However, notice that this relation also applies for assertions (where the illocutionary effect is the performative update with a guarantee by the speaker for the asserted proposition), and *hereby* never occurs with assertions. This fact can be captured if we assume that the causal or instrumental relation holds to the proposition of the speech act, the TP: It indicates that the proposition becomes true with the help of the locutionary act. For in the case of an assertion, the proposition is true independent of the locutionary act.

One observation that supports this analysis is that *hereby* can occur in embedded clauses that do not have their own speech-act potential, as observed by Lee (1975):

(44) $$I\,regret \, that\,I\,have \, to \, inform \, you \, that \, you \, are \, hereby \, fired.$$

We assume that *hereby* just indicates that its host proposition expressed by [_TP_ *you are layed off*] becomes true as a result of the locutionary act expressed by (44) as a whole, which is itself a declaration.^21^ Becoming true as a result of an event itself is a complex notion, involving causality, that we will not go into here. But one minimal requirement is that the proposition is not true *before* the causing locutionary act. This rules out assertions, as speakers that commit to the truth of a proposition should have evidence that this proposition is true, which means that the proposition should already be true at the point of this commitment.^22^

Notice, also, that *hereby* in this analysis is not really self-referential; it is part of the locutionary act, and when interpreted, refers to that event. This is possible if the locutionary act and the illocutionary act are interpreted in succession, bound together by dynamic conjunction.

## Conclusion

In this paper we have investigated the nature of performative speech acts, by concentrating on the technical issue of how they can be modeled in dynamic semantics. Based on the groundbreaking work of Szabolcsi (1982), we have defended a notion of common ground update that does not add to a body of information *about* the world, but induces *changes* of the world. We have seen how explicit performative sentences like *I (hereby) congratulate you* can be analyzed, but also implicit performatives like *The meeting is (hereby) adjourned.* Assertions, we argued, also contain a performative component, in which the speaker issues a guarantee for the truth of a proposition. The informative aspect of assertions comes about through its intended perlocutionary effect. We have sketched how a number of other speech acts, like commissives, directives, exclamatives, optatives and definitions, may be analyzed in this general framework.

We have also considered how locutionary acts may be modeled as index changes. The utterance event can be seen as a performative change of the common ground. Speech acts can be seen as a combination of a locutionary act, an utterance by a speaker to an addressee, and a subsequent illocutionary act that leads to a change in the social world, broadly speaking. With assertions, there is an additional primary perlocutionary act that proposes an informative update with the asserted proposition, which depends on the reactions of the addressee.

Finally, we discussed the deictic adverbial *hereby* as a causal link between the locutionary act and the truth of the core proposition of the illocutionary act. We have identified as a desideratum the integration of a model for discourse referents that can handle, among other things, anaphoric reference to the speech acts that the discourse itself consists of.

## Footnotes

1 There is also the approach of Searle & Vanderveken (1985) and Vanderveken (1990) that is intended to cover a wide range of speech acts in a non-dynamic setting. In this framework, performative utterances are represented by formulas that themselves are true or false, which does not capture their act-like nature.

2 Lascarides and Asher (2003) define an action that makes a proposition true as a function from worlds to worlds; however, they do not give any restriction except that the proposition should hold at the output world. A similar index change is proposed in Hunter et al. (2018), rule 21.

3 Alternatively, we could define a version of functional update that is restricted to those indices i for which φ(i) is false. However, there are many speech acts that can be performed multiple times, either because the speaker suspects that previous performances were unsuccessful, or in order to increase the overall force of the speech act, e.g. with congratulations.

4 The proposed reconstruction of informative update and performative update corresponds to the analysis of Searle (1976) as to the direction of fit: In representative speech acts, the words are supposed to follow the world, whereas in declarative speech acts, the world is supposed to follow the words.

5 Szabolcsi (1982) worked within the framework of Montague (1973), where indices are the product of worlds and times W × T, with a temporal order ≤ on the set T of times. Frameworks of branching time can be seen as a simplified version of Montague’s framework: They do not distinguish between word-time pairs <w,t> and <w′,t> iff for all the predecessors t′ ≤ t, the interpretation of every expression for <w,t> and <w′,t′> are the same (cf. Thomason 1984).

6 The choice between functional index change and relational index change is reminiscent of two prominent treatments of counterfactional conditionals. For the semantics of *if φ were the case then ψ would be the* case, interpreted at index i, Stalnaker (1968) proposes access to the unique index i′ that is as similar to i except that φ holds, whereas Lewis (1973) argues that this index is not unique, and allows for a set of such indices.

7 However, there are subtle differences between the use of *hereby* in English and *hiermit* in German. It appears that the former is restricted to a legal register, which makes (17) less felicitous than its German counterpart, *Bürger können hiermit ab sofort Auslandsreisen beantragen.* Also, *hiermit* can be used in imperatives, as in *Verlassen Sie hiermit sofort diesen Raum*, in contrast to *hereby*, cf. **Leave hereby this room immediately.*

8 This analysis has predecessors in philosophy and in linguistics. The operator • is a particular instance of the illocutionary operator F in Searle (1969), where speech acts are generally assigned the structure F(p), where p is a proposition. But different from that, or the more elaborate versions of illocutionary operators in Searle & Vanderveken (1985), the application of this operator does not result in a formula that has a truth value but in a function that changes a context set. The category ActP corresponds to assumptions in the so-called performative hypothesis (Ross 1970), such as the Force Phrase in Rizzi (1997) and the SAP (Speaker-Addressee-Phrase) in Speas & Tenny (2003).

9 Assuming X-bar syntax, a standard model of syntactic representation, cf. Jackendoff 1977, the operator • would be analyzed as the syntactic head of the ActP as below. This structure would trigger additional movements, e.g. of the finite verb *is* to the head of the ActP and of the subject to the specifier of the ActP, which is neglected here. [_ActP_ [_Act′_ [_Actº_ •] [_TP_ [_DP_ *citizens*] [_T′_ [_Tº_ *can*] [*apply for travel abroad*]]]]].

10 Explicit performatives also come with preconditions like (22), except that everyone in the speech community can congratulate, swear, promise – or assert, the speech act we will discuss in the following section.

11 It is well-known that the opposite does not hold. There are predicates that describe communicative acts that cannot be used performatively, such as *insult* (cf. Austin 1962), manner-of-speaking verbs like *whisper*, or verbs like *insinuate* or *lie*.

12 Fortuin (2019) discusses potential counterexamples (Mongolian, Chichewa), but the progressives there may not be standard progressives but have additional meaning components, such as futurate meanings and expressing a focus on the utterance time.

13 For Slovenian, imperfectives tend to be used in a more formal settings, like swearing in as a judge, and some verbs, like ‘congratulate’, only allow for imperfectives (Močnik 2015).

14 In my own fieldwork on the Oceanic language Daakie (Ambrym, Vanuatu) I identified another marking of performatives. The language distinguishes between Realis and Irrealis modality, where Realis is used for events and states that are realized in the past of or at the index of utterance. In performative utterances, irrealis is used, which is predicted as realis would presuppose that the new states obtains already at the index of utterance.

15 Following assumptions of X-bar theory, the structure would be, more specifically: [_ActP_ [_Act′_ [_Actº_ •][_ComP_ [_Com′_ [_Comº_ ⊢] [_TP_ *can apply for travel abroad*]]]]].

16 Moore’s paradox *It is raining but I don’t believe it* can be explained by the oddness of guaranteeing for the truth of a proposition, accepting social sanctions if it does not hold, and publicly accepting that one does not think that the proposition is true.

17 The distinction between *establishing* the truth of a proposition about one’s future behavior, as in commissives, and of *committing* to a proposition about the truth of one’s future behavior is very subtle, as it is in the speaker’s power to make the proposition true (cf. also Condoravdi & Lauer 2011, who argue that the assertion of a commitment to act in a particular way reduces to the commitment to act in this way). This may explain why this type of speech act is typically not marked by morphosyntactic means (cf. Gärtner & Steinbach 2019; Kaufmann 2019), with rare exceptions like the Korean promissive marker (cf. Pak 2008). But see Gärtner (2020) for a different proposal for the rarity of commissive markers that is based on the fact that in many cases, the promise to act in a certain way may be unnecessary because one can instead act right away, a property that is not shared with directives.

18 We can allow for intermittent times within a branch of development as well. We can also assume that in general, propositions are functions from sets of indices on a branch to truth values, and allow for propositions that map singleton sets of indices. This is not done here to keep notation changes at a minimum.

19 Figure (40) presents things at a certain level of granularity, but of course a more coarse-grained (or even more fine-grained) modelling would be possible. One particularly attractive view is to choose a granularity level that corresponds to the duration of what humans perceive as “now”, in the magnitude of 2–3 seconds (cf. Pöppel 2009), which corresponds to the processing of longer syntactic phrases or prosodic units (cf. Meyer 2018; Vollrath et al., 1992), which in turn are related to individual speech acts (cf. Truckenbrodt 2015). In this way, locutionary acts may help to synchronize time in the common ground during conversation. The representation in (40) would then be too fine-grained, as the states c_2_ and c_3_ can be eliminated.

20 Thanks to Sam Featherston for this observation. For example, *hiermit* can be used with commands, as in *Komm hiermit sofort her!* ‘Come here immediately’, or in deontic statements *Anträge können hiermit ab sofort gestellt warden.* ‘Applications can be submitted as of now’.

21 Eckardt (2012) points out that the position of *hereby* may vary in complex clauses, as in *I (hereby) inform you that you are (hereby) fired.* This is compatible with our approach, as *hereby* may mark that the proposition ‘speaker informs addressee that addressee is fired was not true before the utterance, or alternatively the proposition ‘addressee is fired’ was not true before the utterance.

22 As we have seen in footnote 21, German *hiermit* can express a purely temporal relation. On the suggestion of one reviewer, one can construct cases in which *hiermit* can be used with assertions, e.g. when uttering at 8 pm sharp at looking at one’s watch: *Es ist hiermit acht Uhr*. Notice that in this situation the proposition ‘it is eight o’clock’ was not true before the sentence was uttered.
